# Hypovitaminosis D in patients with oral squamous cell carcinoma: Is a risk factor of developing this neoplasia?

**DOI:** 10.4317/medoral.26692

**Published:** 2024-11-25

**Authors:** Andrea Maturana-Ramiìrez, Juan Aitken-Saavedra, Gabriel Rojas-Zúñiga, Gonzalo Rojas-Alcayaga, Iris Espinoza-Santander, Antonia Rebolledo, Rodrigo Fuentes, Montserrat Reyes-Rojas, Cristóbal Araya, Diego Lazo, Egardo Caamaño

**Affiliations:** 1 Department of Oral Pathology and Medicine, Faculty of Dentistry, Universidad de Chile, Santiago, Chile; 2 Therapeutic Diagnostic Center Odontology and Pathological Anatomy Service, Hospital Complex San Jose, Santiago, Chile; 3 Undergraduate, Faculty of Dentistry, Universidad de of Chile, Santiago, Chile; 4 Head and Neck Surgery Service, National Cancer Institute. Santiago, Chile; 5 Medical Technologist. Laboratory of Endocrinology and Reproductive Biology of the Clinical Hospital, University of Chile

## Abstract

**Background:**

Hypovitaminosis D raised a significant public health concern due to its potential association with various diseases, including Oral Squamous Cell Carcinoma (OSCC). The objective of this study was to compare serum 25(OH)D3 levels between individuals with and without OSCC, and by subgroups based on their smoking habits.

**Material and Methods:**

A case-control study was conducted utilizing progressive multicenter recruitment, involving 46 patients with Oral Squamous Cell Carcinoma (OSCC) and 65 controls. Serum levels of 25(OH)D3 were evaluated via electrochemiluminescence. Patients were categorized according to their vitamin D levels into sufficiency, mild deficiency, moderate deficiency, and severe deficiency. Comparative analyses of serum 25(OH)D3 levels were performed between OSCC patients and controls, as well as among subgroups based on their smoking habits. Group comparisons were made with the Mann-Whitney test, and subgroup analyses used the Kruskal-Wallis test. Significance was set at *p* < 0.05

**Results:**

91% of participants, including both OSCC patients and controls, exhibited some degree of 25(OH)D3 deficiency. Among them, 71.7% of OSCC patients and 50.7% of controls had serum levels characterized by moderate to severe deficiencies. Patients with OSCC showed lower levels of 25(OH)D3, with medians of 20.2 ng/ml (IQR 9.48), compared to controls, with medians of 24.8 ng/ml (IQR 9.13) (*p*=0.002). Furthermore, when comparing the medians among the four study groups (smoking and non-smoking controls and smoking and non-smoking patients with OSCC), a significant difference was observed between non-smoking control patients with 25.04 ng/ml (IQR = 9.71) and smoking OSCC patients with 19.65 ng/ml (IQR = 12.14) (*p* < 0.05).

**Conclusions:**

Individuals with oral squamous cell carcinoma (OSCC) exhibited lower serum levels of vitamin D (25(OH)D3) compared to controls, suggesting a potential link between vitamin deficiency and the development of this type of cancer. Vitamin D supplementation could serve as a preventive and therapeutic strategy.

** Key words:**Smoker, oral neoplasia, calcitriol, oral squamous cell carcinoma.

## Introduction

The Globocan 2020 report discusses the overarching impact of cancer on premature mortality, highlighting approximately 0.37 million new cases and 0.17 million fatalities attributed to lip and oral cavity cancer (1). OSCC is the most common subtype of HNSCC (Head and neck squamous cell carcinoma (HNSCC)) and is characterized by high rates of metastasis and recurrence and resistance to traditional chemotherapy (2). Carcinogenesis is a complex, multi-step process in which genetic events within signal transduction pathways are subverted/altered resulting in enhancement to the cell's ability for proliferation, uncontrolled apoptosis or growth by invading locally or metastasizing to distant sites (3). Activation of proto-oncogenes and/or inhibition of tumor suppressor genes by environmental factors, such as smoking and infections, may heighten the risk of OSCC (4).

The primary associated risk factors of OSCC include the synergistic use of tobacco and alcohol (2). Among the factors potentially increasing susceptibility to OSCC development, systemic factors like hypovitaminosis must be considered. The vitamin D regulates numerous cellular pathways that could have a role in determining cancer risk and prognosis (5). Vitamin D can be obtained from exposure to sunlight and through the consumption of certain foods. After intake, it undergoes conversion primarily in the liver to form the main circulating form called 25-hydroxyvitamin D (25(OH)D), and then further processing occurs in the kidneys to produce the active form, 1,25-dihydroxyvitamin D. The most reliable way to evaluate someone's vitamin D levels is by measuring the concentration of 25(OH)D in their blood plasma (6).

Adequate calcitriol levels are linked to a reduced risk of development and progression of various cancers due to its antitumor effects (7). In vitro and ex vivo models of oral dysplasia treated with 1,25-(OH)2D3, showed decreased levels of nuclear β-catenin, promoted membranous localization of E-cadherin and nuclear localization of vitamin D receptor (VDR) and, the cells diminished cell migration and viability *in vitro* (8). In the same way, a decrease in cell proliferation was demonstrated in OSCC cell lines through the use of 1,25-(OH)2D3 in an *in vitro* model (9). On the other side, there is a proposition that vitamin D could modify the carcinogenicity of tobacco chemicals (10), a proposition of particular relevance given that tobacco remains one of the risk factors most strongly associated with OSCC (11). This pioneering study in a Latin American population aims to compare vitamin D (25(OH)D3) levels between patients with OSCC and controls, taking into account smoking habits. The results obtained may prove fundamental for the chemoprevention of this disease.

## Material and Methods

- Design and sample

This multicenter case-control study involved two groups of participants aged 18 and above: one with Oral Squamous Cell Carcinoma (OSCC) and a control group. Each group was subdivided into smokers and non-smokers. The sample size, comprising 46 cases and 65 controls, was determined with a significance level of 5% and a desired statistical power of 0.9, making it both adequate and significant for the study's objectives. To be eligible for inclusion, case patients had to receive a histological diagnosis of OSCC from a specialist in Oral Pathology, following the 2017 WHO criteria (12). These patients were sourced from the Oral Medicine Clinic of the Faculty of Dentistry at the University of Chile and the North Metropolitan Health Service in Santiago de Chile, specifically the San José Hospital and the National Cancer Institute. The control group consisted of individuals over 18 years of age without OSCC or Potentially Malignant Oral Disorders (PMOD). In this study, patients with OSCC and controls were recruited from health centers within the same geographical area in the city of Santiago. Additionally, all patients were part of the Chilean public healthcare system. Clinical evaluations were conducted by a specialist in Oral Pathology. Exclusion criteria encompassed patients irradiated in the head and neck area, those with terminal illnesses, severe neurological damage, mental disorders, liver or kidney failure, pregnancy, immunological diseases, or those who had taken vitamin D supplements in the past 6 months. Ethical approval for the research project was obtained from the Ethics Committee of the North Metropolitan Health Service in Santiago of Chile (063/2019, august 2019), and the study adhered to the recommendations of the Declaration of Helsinki (13). All participants provided written informed consent before their involvement in the study.

- Determination of serum vitamin D level (25(OH)D3 levels

All patients underwent serum calcidiol (25(OH)D3) level measurements, conducted at the Clinical Laboratory of the Clinical Hospital of the University of Chile. A fasting blood sample (minimum 1 mL, with a minimum serum volume of 500 µL) was obtained and analyzed in the Endocrinology Laboratory of the same hospital. The electrochemiluminescence method, employing a standardized technique on the Cobas 601 equipment (ROCHE HITACHI), was used for analysis. The classification of 25(OH)D3 levels was as follows: Sufficiency (>35 ng/ml), Mild deficiency (25-35 ng/ml), Moderate deficiency (12.5-24.9 ng/ml), and Severe deficiency (<12.5 ng/ml) (6).

- Determination of smoking habit

The cigarette-smoking status of participants was assessed through a self-administered questionnaire. None of the study participants reported using electronic cigarettes or smokeless tobacco products. Smoking status was categorized in the questionnaire as follows: 'smoker,' defined as an individual who had smoked at least 100 cigarettes in total since initiation of smoking, and 'nonsmoker,' defined as an individual who had either never smoked or had smoked fewer than 100 cigarettes in total since initiation of smoking (14). No patient smoked electronic cigarettes. The pack-year index (PYI) was used, calculated by multiplying the number of cigarettes smoked per day by the number of years, divided by 20. The following classification was adopted: PAI 0 for non-smokers; Level 1 smoker, PYI ≤ 28; Level 2 smoker, PYI > 28 (15).

- Statistical analysis

Descriptive statistics were performed using the median and Interquartile range. The distribution of the data was determined using the Shapiro-Wilk test. Two-group comparisons utilized the Mann-Whitney test, while subgroup comparisons (smoking and non-smoking control patients, and smoking and non-smoking OSCC patients) employed the Kruskal-Wallis test. To isolate the group or groups that differ from the others, a multiple comparison procedure was used (Dunn's method). To investigate the differences between categorical variables within the same population (gender and smoking habits based on the presence or absence of an OSCC diagnosis) and to compare the various categories of 25(OH)D3 levels between case patients and controls, a Chi-squared test was performed. Differences were considered significant with a probability of error *p*<0.05. All analyses were carried out using STATA 15.0 software.

## Results

The study included 46 patients with Oral Squamous Cell Carcinoma (OSCC) and 65 controls. Among the OSCC patients, 61% were men, 21.5% were women, and the average age was 62.8 years (SD:14.22). The tongue was the most frequent location of OSCC (52.2%), followed by ridge/gingiva (19.6%). The most observed clinical presentations of OSCC were ulcers (54.4%) and tumors (37%). Of the 46 patients with OSCC, 20 died, with an average post-diagnosis survival time of 13.95 months. Among the 24 living patients, 22 were diagnosed less than 5 years ago, and only 2 were diagnosed more than 5 years ago. Detailed clinicopathological characteristics of OSCC patients and controls can be found in Table 1.

- 25(OH)D3 levels according to diagnosis

Only 2 patients with Oral Squamous Cell Carcinoma (OSCC) (4.4%) and 8 controls (12.3%) had sufficient serum levels of 25(OH)D3. A percentage of 91% of the remaining patients, including both OSCC patients and controls, exhibited some degree of 25(OH)D3 deficiency. Specifically, 11 OSCC patients (24%) and 24 controls (37%) had mild deficiencies, while 25 OSCC patients (54.3%) and 32 controls (49.2%) had moderate deficiencies. Finally, 8 OSCC patients (17.4%) and 1 control (1.5%) were found to have severe deficiencies. A chi-squared test was conducted and was significant (*p* < 0.05), indicating that the proportions are different across all categories.

**Figure 1:**
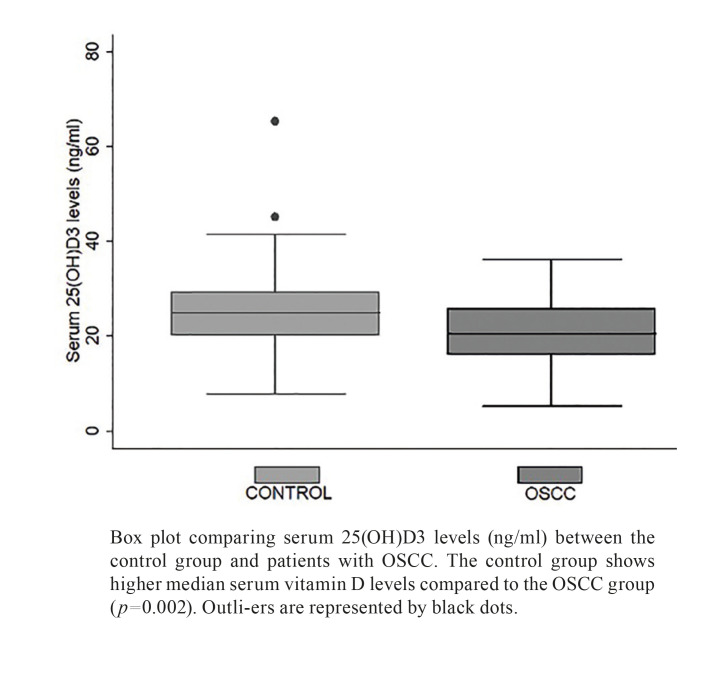
Serum 25(OH)D3 levels in control and OSCC patients.

Patients with OSCC exhibited lower levels of 25(OH)D3, with a median of 20.2 ng/ml (IQR 9.48), compared to control subjects, who had a median of 24.8 ng/ml (IQR 9.13) (*p*=0.002) (Fig. 1). The distribution of 25(OH)D3 levels by sex revealed a median of 24.4 ng/ml (IQR 9.8) in men and 19.65 ng/ml (IQR 9.2) in women with OSCC, whereas in the control group, men had a median of 24.4 ng/ml (IQR 7.38) and women had a median of 25.2 ng/ml (IQR 10.9). Comparisons between 25(OH)D3 levels between men and women in OSCC and control groups showed no statistically significant differences (*p*>0.05).

Regarding the degree of differentiation of OSCC, among the 46 cases, 1 was poorly differentiated (19.05 ng/mL), 16 were moderately differentiated (20.8 ng/mL), and 28 were well differentiated (19.43 ng/mL). There were no statistically significant differences in Vitamin D levels between these subgroups.

- 25(OH)D3 levels, according to smoking habit

**Figure 2:**
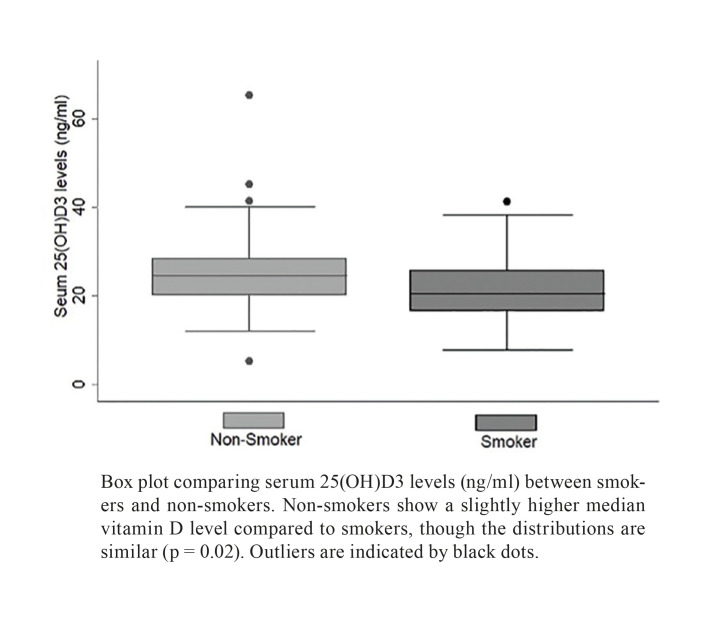
Serum 25(OH)D3 levels in smokers and non-smokers.

**Figure 3:**
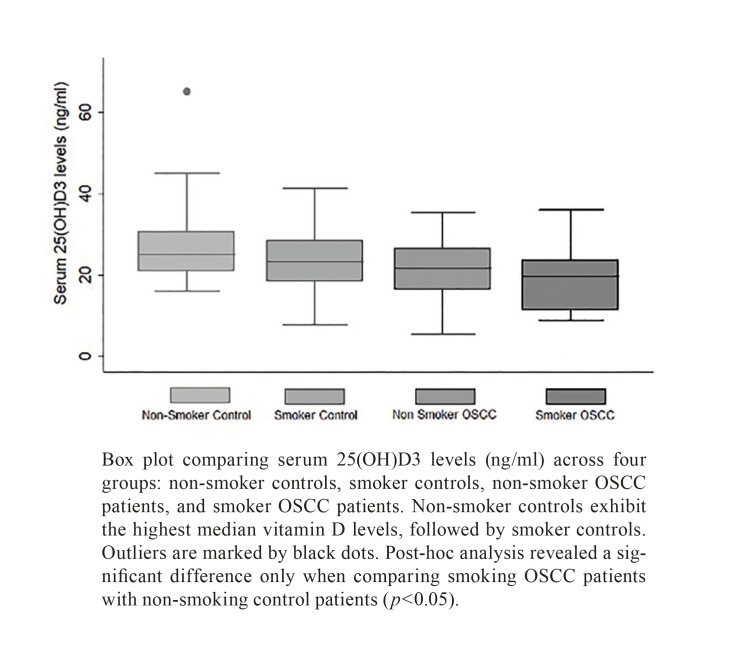
Serum 25(OH)D3 levels by smoking status and oscc diagnosis.

In the examined group, 47.8% of patients diagnosed with OSCC and 30% of the control subjects reported being smokers. Among individuals who smoked, regardless of OSCC diagnosis, the median level of 25(OH)D3 stood at 20.4 ng/ml (IQR = 9.22), while non-smokers exhibited a higher median level of 24.43 ng/ml (IQR = 8.3) (*p* = 0.02) (Fig. 2). For each group, the median levels of 25-hydroxyvitamin D (ng/ml) and interquartile ranges (IQR) were as follows: Non-smoking controls: 25.04 ng/ml (IQR = 9.71); Smoking controls: 23.3 ng/ml (IQR = 10.1); Non-smoking patients with OSCC: 21.6 ng/ml (IQR = 10.14); and Smoking patients with OSCC: 19.65 ng/ml (IQR = 12.14) (Fig. 3) (*p*=0.004). Post-hoc analysis using Dunn's Method for pairwise multiple comparisons revealed a significant difference only when comparing smoking OSCC patients with non-smoking control patients (*p*<0.05).

## Discussion

The aim of this study was to compare serum 25(OH)D3 levels among individuals with and without OSCC, considering their smoking habits. Regarding the sample characterization, our study observed a male predominance in OSCC cases, consistent with existing literature where men represent approximately 70% of cases (2). The age distribution of affected individuals and the predominant anatomical site (the tongue being the most common location for OSCC) observed in our study align with findings from prior research (16).

Although hypovitaminosis of 25(OH)D3 was prevalent among the majority of patients in our study, significant differences were noted between OSCC patients and controls regarding serum 25(OH)D3 levels. Similar findings have been reported in India, where OSCC patients showed serum 25(OH)D3 levels of 18.15 ng/ml compared to controls with 34.16 ng/ml (17), albeit using a different measurement methodology. In our study, 71.8% of patients with OSCC presented serum levels with moderate and severe deficiencies, which coincides with previous reports that indicate a prevalence for these same ranges ranging between 74.5% (18) and 100% (19) among OSCCs patients. It is important to note that another study did not find significant differences in 25(OH)D levels between OSCC patients and control subjects (20). However, unlike our results, that study also did not find differences in tobacco consumption between cases and controls. This discrepancy could be due to the fact that tobacco consumption may decrease 25(OH)D levels.

Lower levels of 25(OH)D3 have been linked to an elevated risk of OSCC and increased mortality rates (18,21). Conversely, some studies suggest that a higher dietary intake of vitamin D is associated with a reduced risk of oral and pharyngeal cancer, particularly among smokers and individuals with severe alcohol consumption (22), while others propose a weak or unlikely association between vitamin D and the risk of oral and pharyngeal cancer (23). Vitamin D deficiency is associated with an increased risk of cancer, disease progression, reduced survival rates, higher rates of recurrence, and adverse reactions to chemotherapy (21). Elevated Vitamin D Levels from Diet, Genomic Variations, and Circulating 25-OHD May Confer Protection Against Head and Neck Cancer and Enhance Prognosis in Patients. Implications for Reducing Incidence and Mortality Requiring Further Investigation (24). It has been hypothesized that hypovitaminosis D may elevate the risk of developing OSCC from an OPMD, impacting immune responses (25). This deficiency is believed to be linked to an increased risk and progression of cancer, decreased survival rates, higher recurrence rates, and increased adverse reactions to chemotherapy (26). Hypovitaminosis was prevalent among the vast majority of patients in our study. Inadequate levels of vitamin D, known as hypovitaminosis D, can result in various skeletal and non-skeletal consequences. The elderly population is particularly vulnerable to vitamin D deficiency because aging alters both the synthesis and processing of vitamin D. This is often due to factors such as decreased exposure to sunlight and a decline in the skin's ability to produce vitamin D (27). In our study, deficits in the control group exceeded those previously reported (19), possibly attribuTable to various factors such as geographical location, sample size, dietary patterns, and age distribution. Considering the manifold benefits associated with adequate vitamin D levels, its serum evaluation could serve as a preventive measure in this age group. Regarding smoking, our study found that patients with OSCC who smoke had lower vitamin D levels compared to non-smoking controls. A study has underscored an increased risk of tobacco-related cancer among individuals with insufficient levels of vitamin D. It has been suggested that vitamin D might impact the carcinogenicity of tobacco smoke, a factor especially pertinent in our population of OSCC patients, who predominantly smoke. Modulating vitamin D levels could potentially play a role in preventing the development of oral cancer (10). Our study revealed a 15% difference in plasma concentration of 25(OH)D3 between OSCC patients who smoke and smoking control patients, compared to the 7% reported in active smoking patients by (21). This is consistent with a meta-analysis, which evaluated differences in circulating vitamin D levels between smokers and non-smokers and revealed that smokers have lower circulating vitamin D levels than non-smokers (28).

A meta-analysis examining serum inflammatory biomarkers following vitamin D supplementation in cancer patients revealed significant reductions in TNF-α, IL-6, and C-reactive protein (CRP) levels, while IL-10 levels remained unchanged (29). These findings underscore the potential of vitamin D in tumor suppression through the modulation of inflammatory processes. In vitro models have also suggested that vitamin D supplementation inhibits the proliferation of OSCC cancer cells (9). The effects of vitamin D in the context of OSCC could be due to its previously described roles in various antineoplastic activities, encompassing antiproliferative, antimetastatic, antiangiogenic, proapoptotic, and pro differentiation actions. It exerts its effects through molecular pathways such as the modulation of cyclin-dependent kinase (CDK) inhibitors like p21 and p27 (7,9). Moreover, vitamin D deficiency has been associated with the induction of apoptosis by downregulating anti-apoptotic genes like BCL-2 and upregulating pro-apoptotic genes like BAX (7), underscoring its pivotal role in OSCC carcinogenesis, morbidity, and mortality (30).

Limitations of our study include unaccounted variables such as diet, sun exposure, body mass index (BMI), and systemic pathologies associated with low 25(OH)D3 levels (e.g., diabetes mellitus and cardiovascular diseases) that may influence our results. Although the difference in the distribution of men and women between cases and controls in our study could be a confounding variable, the results do not suggest a sex-based difference in vitamin D levels in this study. Future studies with larger sample sizes should explore subgroups and establishing consistent definitions for considering 25(OH)D3 deficiency is required. Furthermore, it is necessary to incorporate randomized clinical trials with long-term follow-up and investigate specific genetic polymorphisms of the vitamin D receptor (VDR) in people with OSCC and in OPMD, which can help develop more precise and effective chemopreventive and therapeutic strategies. in the treatment.

Given its antineoplastic effects, vitamin D supplementation in patients with hypovitaminosis could be considered as an adjuvant strategy in the treatment of patients with OSCC and potentially chemopreventive in patients with OPDM developed countries. Considering the beneficial effects of vitamin D and the carcinogenic effects of tobacco, our results support the evaluation of serum vitamin D levels and its supplementation as an appropriate strategy in patients with OSCC, in smokers, and in patients at risk of malignant transformation, such as those with OPMD.

## Figures and Tables

**Table 1:** Characteristics of patients with OSCC and controls.

Characteristics		Cases n (%)	Controls n (%)
Sex	Male	28 (60.9)	14(21.5)
	Female	18 (39.1)	51(78.5)
Age group	<45 years	6 (13.0)	5 (7.7)
	45 years or older	40 (87.0)	60 (92.3)
Tumor location	Tongue	24 (52.2)	-
	Ridge/gum	9 (19.6)	-
	Cheek	7 (15.2)	-
	Floor of mouth	5 (10.9)	-
	Labial mucosa	1 (2.1)	-
Clinical appearance	Ulcer	25 (54.3)	-
	Tumor	17 (37.0)	-
	Plaque	4 (8.7)	-
Categorization by 25(OH)D3 levels	Sufficiency	2 (4.3)	8 (12.3)
	Mild	11 (24.0)	24 (37.0)
	Moderate	25 (54.3)	32 (49.2)
	Severe	8 (17.4)	1 (1.5)
Categorization by pack-year index	Level 0 (PYI = 0)	24(52.2)	41 (63.0)
	Level 1 (PYI ≤ 28)	15 (32.6)	20 (30.8)
	Level 2 (PYI > 28)	7(15.2)	4 (6.2)
Total		46 (100)	65 (100)
